# Mastery Imagery Ability Is Associated With Positive Anxiety and Performance During Psychological Stress

**DOI:** 10.3389/fpsyg.2021.568580

**Published:** 2021-03-29

**Authors:** Sarah E. Williams, Mary L. Quinton, Jet J. C. S. Veldhuijzen van Zanten, Jack Davies, Clara Möller, Gavin P. Trotman, Annie T. Ginty

**Affiliations:** ^1^School of Sport, Exercise and Rehabilitation Sciences, University of Birmingham, Birmingham, United Kingdom; ^2^The Wright Institute, Berkeley, CA, United States; ^3^Department of Psychology and Neuroscience, Baylor University, Waco, TX, United States

**Keywords:** anxiety direction, cognitive anxiety, mastery imagery, psychological stress, somatic anxiety

## Abstract

Mastery imagery (i.e., images of being in control and coping in difficult situations) is used to regulate anxiety. The ability to image this content is associated with trait confidence and anxiety, but research examining mastery imagery ability's association with confidence and anxiety in response to a stressful event is scant. The present study examined whether trait mastery imagery ability mediated the relationship between confidence and anxiety, and the subsequent associations on performance in response to an acute psychological stress. Participants (*N* = 130; 55% male; *M*_*age*_ = 19.94 years; *SD* = 1.07 years) completed assessments of mastery imagery ability and engaged in a standardized acute psychological stress task. Immediately prior to the task, confidence, cognitive and somatic anxiety intensity, and interpretation of anxiety symptoms regarding the task were assessed. Path analyses supported a model whereby mastery imagery ability mediated the relationship between confidence and cognitive and somatic anxiety interpretation. Greater mastery imagery ability and confidence were both directly associated with better performance on the stress task. Mastery imagery ability may help individuals experience more facilitative anxiety and perform better during stressful tasks. Improving mastery imagery ability by enhancing self-confidence may help individuals successfully cope with anxiety elicited during stressful situations.

## Introduction

Events in life that require optimal performance of the individual are often associated with increased feelings of psychological stress (e.g., sporting competition, job interview, giving a speech in class). Experiences of such stress can negatively impact not only our cognitions and emotions (e.g., feelings of anxiety), but also how we perform (Lazarus et al., 1952; Cohen, 1980). Research suggests that perceptions of psychological stress during a task can elicit more negative affect, and hinder performance in exams, negotiating, sporting competition, and other cognitive tasks (Robinson et al., 2013; Jamieson et al., 2016; Crum et al., 2017). Consequently, it is important for research to examine factors associated with more adaptive responses to stress, better performance.

Anxiety is one of the most frequently experienced emotions during and in anticipation of acute psychological stress exposure. Symptoms of anxiety can be classified as cognitive or somatic, making it a multidimensional construct. Cognitive anxiety is the mental component of anxiety, and includes negative thoughts, concerns, and worries (Morris et al., 1981; Martens et al., 1990). Somatic anxiety refers to the bodily symptoms and sensations experienced (Martens et al., 1990), such as increases in heart rate, perspiration, and respiration. Both cognitive anxiety and somatic anxiety are typically elevated when exposed to an acute psychological stress (Williams et al., 2017) and can account for a large proportion of variance in performance during stressful situations (Seipp, 1991). Although it is overly simplistic to assume that anxiety is always detrimental to performance, a number of studies have demonstrated that higher levels of anxiety tend to be associated with poorer performance (Weinberg and Genuchi, 1980; Hopko et al., 2003; Vitasari et al., 2010; Moore et al., 2013). As such, anxiety is an important emotion to study when examining factors associated with better performance.

As well as anxiety intensity, the interpretation of anxiety symptoms (i.e., whether an individual perceives that the anxiety they are experiencing will have a positive or negative effect on the situation) can impact how individuals cope or perform when in stress evoking conditions (Jones and Swain, 1995; Swain and Jones, 1996; Chamberlain and Hale, 2007; Carrier et al., 2014). For example, some athletes frequently report experiencing high levels of anxiety that they believe are facilitative to performance (Hanton and Jones, 1999). In academic settings, positive interpretations of anxiety symptoms have been associated with better note-taking performance (Carrier et al., 2014). Interestingly, research has shown that at times it is the interpretation of anxiety symptoms (also referred to as anxiety direction), rather than the intensity, that can be a stronger predictor of performance (Chamberlain and Hale, 2007; Neil et al., 2012). Specifically, interpreting anxiety symptoms as more facilitative predicts better performance (Chamberlain and Hale, 2007; Neil et al., 2012). Therefore, it is important to examine both the intensity and interpretation of anxiety experienced.

One disposition thought to influence both the intensity and interpretation of anxiety experienced, particularly during psychologically stressful situations, is confidence. Studies have demonstrated that in a given situation, greater confidence is associated with lower levels of both cognitive and somatic anxiety (Neil et al., 2012; Williams and Cumming, 2012b, 2015; Quinton et al., 2018). Additionally, higher levels of confidence have also been proposed to buffer or protect against the negative effects of anxiety (Jones and Hanton, 2001; Hanton et al., 2004). Specific to stress, confidence, or its situational specific form self-efficacy, has been identified as a key antecedent in both the appraisal of stress, and the subsequent emotional and behavioral responses experienced (Blascovich and Mendes, 2000; Skinner and Brewer, 2004; Jones et al., 2009). For example, Neil et al. (2012) demonstrated that greater confidence experienced prior to line-out throws in rugby was associated with lower levels of anxiety and more facilitative interpretations of these symptoms. Greater confidence and more facilitative anxiety were also associated with better performance (Neil et al., 2012). Feeling more confident about an upcoming stressful event is therefore likely to be associated with lower levels of anxiety, more facilitative interpretations of these symptoms, and subsequently lead to better performance. However, it is important to establish how stable traits or dispositions play a role in the relationship between confidence, anxiety, and performance in response to acute psychological stress.

Mental imagery is a technique that is frequently employed in the regulation of confidence and anxiety (Cumming and Williams, 2013). Specifically, using images of feeling confident and in control of difficult situations appears to instill greater confidence and regulate anxiety by lowering levels, and/or eliciting more positive perceptions of anxiety symptoms in response to stress evoking situations (Cumming et al., 2007; Mellalieu et al., 2009; Williams et al., 2010, 2017). While the effect of using this type of mastery imagery content (i.e., imagery use) on confidence and anxiety is well-established, research has started to examine the importance that an individuals' propensity to image such content (i.e., mastery imagery ability) has on the confidence-anxiety relationship.

Imagery ability can influence the effectiveness of an imagery intervention, with greater benefits typically being experienced by more proficient imagers compared to their lower level counterparts (Robin et al., 2007; Williams et al., 2013). However, this evidence is predominantly from movement-based imagery content to improve performance. More recent research has examined the ability to image content associated with emotions and cognitions, specifically, its correlates and the impact such an ability can have on using this type of imagery content to regulate arousal and anxiety. Findings demonstrated that mastery imagery ability is an important correlate of confidence and anxiety, and appears to influence the impact of stress evoking imagery scripts (Quinton et al., 2019). Mastery imagery ability is positively associated with greater trait confidence (Williams and Cumming, 2012b, 2015; Quinton et al., 2018). Furthermore, Williams and Cumming (2015) demonstrated that through trait confidence, mastery imagery ability was associated with lower levels of trait cognitive and somatic anxiety and more facilitative interpretations of these symptoms. Consequently, individuals who find it easier to image themselves being in control and persevering in difficult and challenging situations, tend to feel more confident in themselves, and have lower levels of trait anxiety and view their anxiety levels as more facilitative.

To further understand the relationship between mastery imagery ability, confidence, and anxiety, Quinton et al. (2018) examined the extent to which mastery imagery ability mediated the relationship between general levels of confidence and cognitive and somatic anxiety intensity and interpretation that athletes tend to experience in relation to their sport. Quinton et al. (2018) explained that Bandura (1997) proposed higher levels of self-efficacy are likely to evoke more positive images, and that more confident individuals are likely to have experienced more recent success, meaning that these achievements may be more readily retrieved from memory to facilitate the imagery process (i.e., make it easier to image; Lang, 1979). In support, results demonstrated that greater confidence was associated with better mastery imagery ability, which in turn was associated with lower levels of cognitive anxiety (Quinton et al., 2018). However, confidence also directly predicted cognitive and somatic anxiety intensity, and neither confidence nor mastery imagery ability were associated with cognitive or somatic anxiety interpretation.

Quinton et al. (2018) proposed that some of their non-significant findings could have been due to confidence and anxiety being assessed at a trait level (i.e., athletes reported how confident and cognitively and somatically anxious they typically feel prior to performing and whether these anxiety symptoms are typically viewed as positive or negative with regards to performance). Given that confidence and anxiety can fluctuate over time and differ depending on the nature of the stress-evoking situation (Thomas et al., 2004; Trotman et al., 2018), it is not surprising that more salient relationships did not emerge. However, to date, research has yet to examine whether mastery imagery ability is associated with confidence and anxiety experienced in response to a specific stress-evoking situation, and more specifically, whether the relationship between confidence and anxiety is mediated by mastery imagery ability. Research has also yet to examine whether mastery imagery ability relates to performance, likely through its previously identified relationship with anxiety. Examining these research questions will help establish whether mastery imagery ability could be an effective strategy to help cope with acute psychological stress.

**Figure 1:**
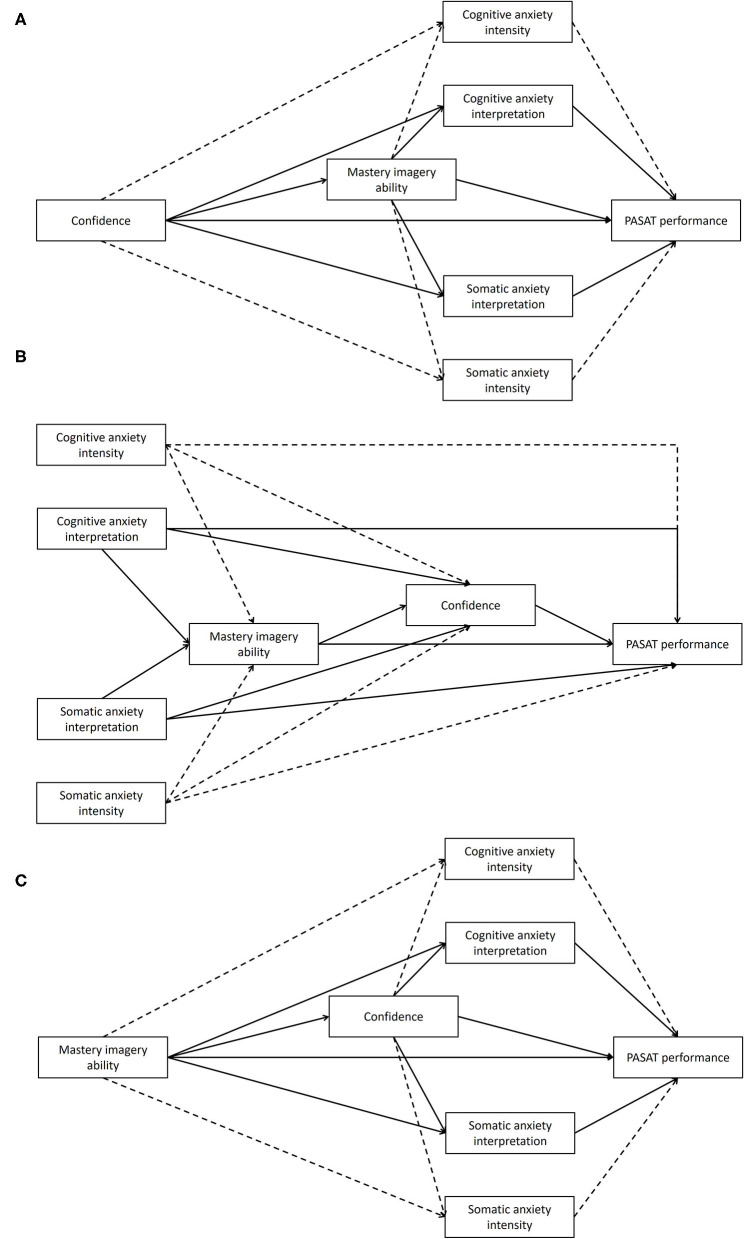
**(A)** Hypothesized model, **(B)** Alternate model 1, **(C)** Alternate model 2. For visual simplicity, controlling variable gender is not presented but was included in the analyses. Full lines represent positive regressions, dashed lines represent negative regressions.

The aim of the present study was to examine whether mastery imagery ability mediated the relationship between confidence and anxiety in response to an acute psychological stress task, and if the anxiety experienced was associated with subsequent performance. Anxiety was assessed in terms of cognitive anxiety intensity, somatic anxiety intensity, cognitive anxiety interpretation, and somatic anxiety interpretation. It was hypothesized that confidence would positively predict mastery imagery ability which would negatively predict cognitive and somatic anxiety intensity, and positively predict cognitive and somatic anxiety interpretation. Therefore, it was proposed that mastery imagery ability would mediate the relationship between confidence and the anxiety constructs. It was also hypothesized that cognitive and somatic anxiety intensity would negatively predict subsequent performance, and that confidence, and cognitive and somatic anxiety interpretation would positively predict performance. Therefore, it was hypothesized that mastery imagery ability would be indirectly associated with performance via anxiety. The hypothesized model was compared to two alternate models to establish whether the hypothesized model was the best fitting model for the data. All three models are displayed in Figure 1.

## Methods

### Participants

Participants were 130 male (*n* = 71) and female (*n* = 59) university students ranging in age from 18 to 25 years old (*M* = 19.94; *SD* = 1.07). Prior to recruitment, the study was approved by the ethics committee at the university where the first author is based and all participants provided written informed consent before taking part in the study. Participants were provided with a course credit upon completion of the study.

### Acute Psychological Stress Task

The 6 min version of the Paced Auditory Serial Addition Test (PASAT; Gronwall, 1977) was completed by all participants. The PASAT is a neuropsychological test. The test is delivered via an audio recording. A series of single-digit numbers between 1 and 9 are played aloud to participants. Participants are required to add together consecutive single-digit numbers and call the answer aloud while remembering the most recent number so it can be added to the next number heard. Thus, the task involves attention and memory as well as simple addition. The numbers were presented at increasing speed every 2 min from 2.0 to 1.6 s and to 1.2 s. To increase the stressfulness of the task, participants were instructed to watch themselves in a mirror. A video camera was placed ≈1 m away and participants were told they would be videotaped so that “body language experts” could assess anxiety levels. Furthermore, participants were also told they would hear a loud beep if they hesitated or said a wrong answer (Veldhuijzen van Zanten et al., 2004). In reality, participants were not videotaped and all participants received the same standardized number of loud beeps every 10 numbers. Participants were also told they began the task with 1,000 points and would lose five points for every incorrect answer. Performance tables showing “top performing” participants were displayed in the laboratory to enhance feelings of stress and competition.

This modified version of PASAT is a standard stress test that has been shown to elicit a substantial stress response (Ring et al., 2002; Veldhuijzen van Zanten et al., 2004; Ginty et al., 2012), and demonstrates good test–retest reliability (Willemsen et al., 1997; Ginty et al., 2013). Additionally, work suggests responses to acute psychological stress in the laboratory generalizes to real life stressors (e.g., Johnston et al., 2008; Zanstra and Johnston, 2011).

### Measures

#### Mastery Imagery Ability

Mastery imagery ability was assessed using the mastery subscale of the Sport Imagery Ability Questionnaire (SIAQ; Williams and Cumming, 2011). The SIAQ consists of fifteen items that assess five different types of imagery ability (skill, strategy, goal, affect, and mastery). The three items that assess mastery imagery content are “Giving 100% effort even when things are not going well,” “Staying positive after a setback,” and “Remaining confident in a difficult situation.” Participants image each item and then indicate on a 7-point Likert type scale how easily they were able to image each item ranging from 1 *(very hard to image)* to 7 *(very easy to image)*. The SIAQ is a valid and reliable measure of imagery ability (Williams and Cumming, 2011). In the present study the mastery subscale demonstrated good internal reliability with the Cronbach alpha coefficient being 0.70.

#### Intensity and Perception of Cognitive Anxiety, Somatic Anxiety, and Confidence

Cognitive anxiety, somatic anxiety, and confidence, and the interpretation of these feelings were assessed using the validated Immediate Anxiety Measures Scale (IAMS; Thomas et al., 2002). Using 7-point Likert type scales, participants first rate the extent to which they feel cognitively anxious (1 = not at all, to 7 = extremely), before indicating whether this feeling is facilitative/positive or debilitative/negative to the upcoming task (−3 = very debilitative/negative, to +3 = very facilitative/positive). This process is repeated for somatic anxiety and confidence but in the present study the data for confidence interpretation was not reported. The IAMS is a valid and reliable measure of state anxiety and confidence (Thomas et al., 2002) and is therefore frequently used to assess anxiety and confidence in response to a stress task (e.g., Moore et al., 2013; Williams et al., 2016).

#### Task Evaluation

Participants rated on separate 7-point Likert type scales how stressful and difficult they found the PASAT and the extent that they were trying to call out the right answers (task engagement). Ratings for all three items were made between 1 (*not at all*) and 7 (*extremely*).

#### Performance

Performance of the PASAT was assessed by all participants beginning the test with 1,000 points. Five points were lost for every incorrect answer the participant provided. Over the 6 min a total of 183 answers were available meaning that possible performance scores ranged from 85 to 1,000, with a higher score indicating better performance.

### Procedure

All participants were tested individually in the laboratory with two researchers present at each session. One researcher administered the questionnaires and psychological stress task while the other scored PASAT performance. Upon arrival to the laboratory participants were informed about the nature of the study and were provided with an information sheet outlining the study details. Those agreeing to take part in the study signed a consent form. As part of a larger study, participants were attached to cardiovascular recording equipment (data not reported) and sat quietly for a 10 min adaptation period during which they provided their demographic information and completed the SIAQ. Next, as part of a larger program of research, participants completed three tasks including the PASAT in a counterbalanced order, each preceded by a 10 min resting baseline period (other tasks not reported in the present study)^1^. After the baseline, participants were read instructions regarding the PASAT and undertook a brief practice to ensure they understood the task. Participants then completed the IAMS with regards to how they felt about the imminent task, immediately followed by the 6 min PASAT. On completion of the stress task, participants rated the task regarding stressfulness, difficulty, and engagement. Finally, all equipment was removed from participants and they were thanked for their participation.

### Data Reduction and Analysis

Analyses were conducted using SPSS version 22 (IBM Corp, USA) and AMOS 22 (IBM Corp, USA). Based on recommendations of Tabachnick and Fidell (2013), data were first inspected for missing data and univariate outliers, before examining multivariate outliers using the Mahalanobis distance at *p* < 0.001. There were no missing data or outliers, therefore all data were retained for the analyses. Mean scores for mastery imagery ability, confidence, cognitive anxiety intensity, somatic anxiety intensity, cognitive anxiety interpretation, somatic anxiety interpretation, PASAT performance, and task stressfulness, difficulty, and engagement were computed. To examine whether the sample found the task stress evoking and difficult, and determine participants were engaged, one-sample *t*-tests were run to compare the sample means of these variables to the midpoint of the scale (4 = somewhat stressful/difficult/engaged). Next, one-way ANOVAs were conducted to examine gender differences in the main variables of interest. Any gender differences were controlled for in all subsequent analyses where appropriate. Regression analysis examined any potential issues with multicollinearity, however, results indicated no such issues.

The hypothesized model was examined using path analyses. Correlations between cognitive and somatic intensity and between cognitive and somatic interpretation were inserted due to the strong association between them (Martens et al., 1990). The chi-squared likelihood statistic ratio (χ^2^; Jöreskog and Sörbom, 1993) was employed to test goodness of fit. Additional fit indices including the Tucker Lewis index (TLI) and comparative fit index (CFI) were employed to reflect incremental fit (values >0.90 and >0.95 indicating an adequate and excellent model fit, respectively; Hu and Bentler, 1999), and the standardized root mean square residual (SRMR; Bentler, 1995) and root mean square error of approximation (RMSEA) were employed to reflect absolute fit (values of ≤ 0.08 and 0.06, respectively, representing an adequate fit; Hu and Bentler, 1999). Mediation analyses was conducted following Hayes' (2013) recommendation of testing for indirect effects. Standardized regressions and 95% confidence intervals, generated from bootstrapping of 2,000 samples, were reported for all significant indirect effects.

Finally, two alternate models were examined using the same analysis procedures as above to compare to the hypothesized model and establish whether the hypothesized model was the best fit for the data. The alternate model 1 examined whether mastery imagery ability mediated the relationship between anxiety and confidence and whether confidence was subsequently associated with performance. Alternate model 2 examined whether mastery imagery ability was associated with confidence that in turn was associated with anxiety, and anxiety in turn associated with performance. Models are displayed in Figure 1.

## Results

### Descriptive Statistics and Gender Differences

Manipulation checks suggest participants found the task to be stressful (*M* = 5.48, *SD* = 1.25) and difficult (*M* = 5.98, *SD* = 0.95). Additionally, participants reported to be engaged in the task (*M* = 6.14, *SD* = 1.04). One-sample *t*-tests showed all three mean scores to be significantly greater than the midpoint of the scale [stressfulness: *t*_(129)_ = 13.44, *p* < 0.001, difficulty: *t*_(129)_ = 23.68, *p* < 0.001, engagement: *t*_(129)_ = 23.45, *p* < 0.001]. Table 1 displays the mean and standard deviations for males, females, and the sample as a whole in the main variables of interest. Separate one-way ANOVAs revealed that males displayed significantly greater mastery imagery ability than females [*F*_(1,128)_ = 17.15, *p* < 0.001, $\eta_{p}^{2}$ = 0.118]. Compared to females, males also reported being significantly more confident [*F*_(1,128)_ = 19.62, *p* < 0.001, $\eta_{p}^{2}$ = 0.133], and they performed significantly better [*F*_(1,128)_ = 20.81, *p* < 0.001, $\eta_{p}^{2}$ = 0.140]. There were no gender differences in any of the anxiety constructs. Gender differences in mastery imagery ability, confidence, and performance were controlled for in all subsequent analyses.

**Table 1:** Total sample, male, and female mean (*M*), and standard deviation (*SD*) values for study variables.

	**Total sample**		**Males**		**Females**	
	***M***	***(SD)***	***M***	***(SD)***	***M***	***(SD)***
Mastery imagery	5.07	1.09	5.41^*^	0.99	4.66	1.06
Confidence	3.48	1.33	3.92^*^	1.32	2.95	1.14
Cognitive intensity	4.38	1.40	4.18	1.52	4.61	1.23
Somatic intensity	3.48	1.44	3.45	1.37	3.53	1.52
Cognitive interpretation	0.74	1.40	−0.69	1.39	−0.80	1.41
Somatic interpretation	−0.64	1.20	−0.61	1.20	−0.68	1.21
PASAT Performance	579.33	124.24	621.52^*^	118.73	528.56	112.47

* *Significantly greater than female means, p < 0.001*.

### Path Analyses

The hypothesized model displayed in Figure 1 was tested by including regressions paths from confidence to mastery imagery ability, from mastery imagery ability to all anxiety constructs (i.e., cognitive anxiety intensity, somatic anxiety intensity, cognitive anxiety interpretation, and somatic anxiety interpretation), and from all anxiety constructs to PASAT performance. Direct regression paths were also included from confidence to all anxiety constructs, and from both confidence and mastery imagery ability to PASAT performance.

**Figure 2:**
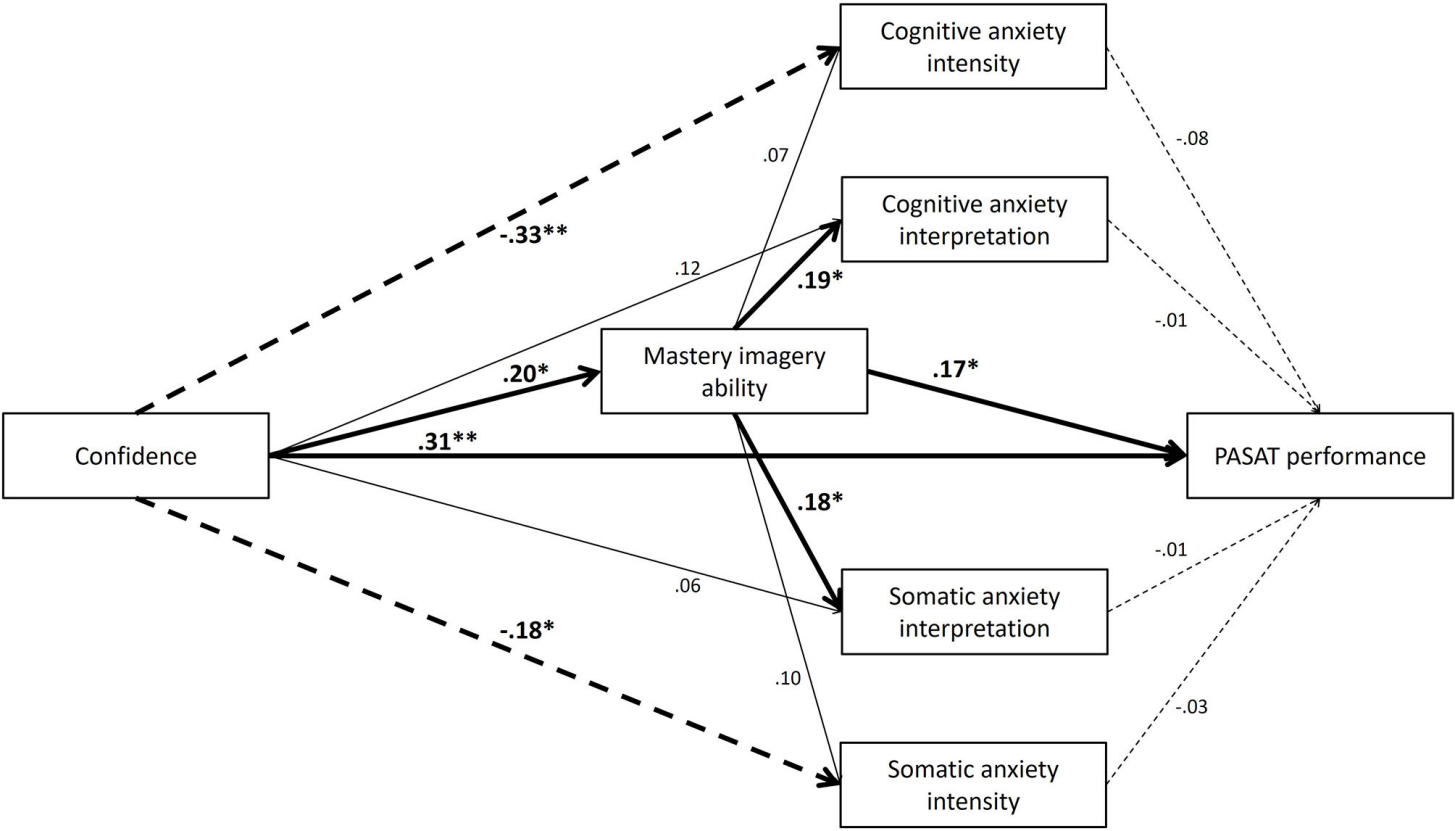
Final model of mastery imagery ability mediating the relationship between confidence and cognitive and somatic anxiety interpretation. All values are standardized coefficients. **p < 0.001, *p < 0.05. For visual simplicity, controlling variable gender is not presented but was included in the analyses. Full lines represent positive regressions, dashed lines represent negative regressions.

The model revealed a good fit to the data, $\chi_{\left(8\right)}^{2}$ = 6.10, *p* = 0.636, CFI = 0.99, TLI = 1.00, SRMR = 0.03, RMSEA < 0.001 (90% CI < 0.001–0.09). As shown in Figure 2, more confident individuals displayed greater levels of mastery imagery ability, that in turn was associated with more positive perceptions of both cognitive and somatic anxiety interpretation. Although confidence was not directly associated with cognitive and somatic anxiety interpretation, there was an indirect pathway via mastery imagery ability (cognitive: *β* = 0.038, *p* = 0.039, 95% CI = 0.001–0.108; somatic: *β* = 0.036, *p* = 0.038, 95% CI = 0.001–0.109). These findings suggest the relationship between confidence and the interpretation of anxiety experienced in response to a psychological stress is mediated by mastery imagery ability. There were no associations between mastery imagery ability and cognitive and somatic anxiety intensity demonstrating that mastery imagery ability did not mediate the relationship between confidence and anxiety intensity. Instead confidence was directly and positively associated with cognitive and somatic anxiety intensity. There were also direct positive associations from both confidence and mastery imagery ability to PASAT performance.

### Alternate Models

**Figure 3:**
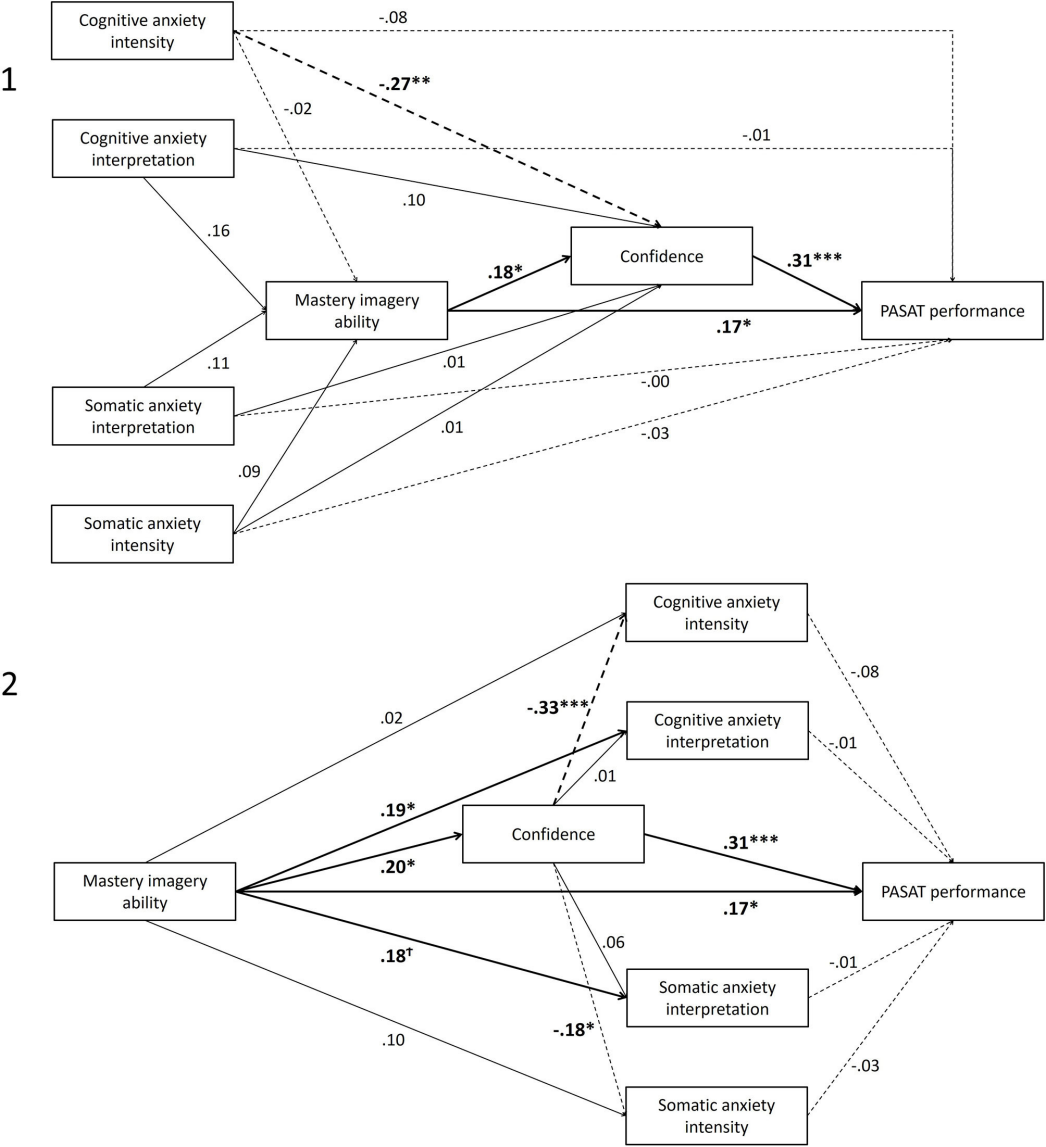
Results of alternate model 1 and alternate model 2. All values are standardized coefficients. ***p < 0.001, **p < 0.01, *p < 0.05, ^†^*p* = 0.05. For visual simplicity, controlling variable gender is not presented but was included in all analyses. Full lines represent positive regressions, dashed lines represent negative regressions.

Alternate model 1 was tested by including regressions paths from all four anxiety constructs to mastery imagery ability, from mastery imagery ability to confidence, and from confidence to PASAT performance. Direct regression paths were also included from all anxiety constructs to confidence, and from the anxiety constructs and mastery imagery ability to PASAT performance. The model revealed a similar fit to the data to the hypothesized model, $\chi_{\left(8\right)}^{2}$ = 8.513, *p* = 0.385, CFI = 0.99, TLI = 0.99, SRMR = 0.04, RMSEA = 0.02 (90% CI < 0.001–0.11). However, as shown in Figure 3, none of the anxiety constructs were significantly associated with mastery imagery ability. Furthermore, only cognitive anxiety intensity was significantly associated with confidence. Consequently, mastery imagery ability did not mediate the relationship between anxiety and confidence, or anxiety and performance.

Alternate model 2 examined whether confidence mediated the relationship between mastery imagery ability and anxiety constructs. Regression paths from mastery imagery ability to confidence, from confidence to all four anxiety constructs, and from all four anxiety constructs to PASAT performance were inserted. Direct regression paths were also included from mastery imagery ability to anxiety constructs, and from mastery imagery ability and confidence to PASAT performance. The model revealed an almost identical fit to the data to the hypothesized model, $\chi_{\left(8\right)}^{2}$ = 6.10, *p* = 0.636, CFI = 0.99, TLI = 1.00, SRMR = 0.03, RMSEA < 0.001 (90% CI < 0.001–0.09). However, as shown in Figure 3, although mastery imagery ability was related to confidence, confidence was not associated with cognitive and somatic anxiety interpretation meaning the confidence did not mediate the direct relationship between greater mastery imagery ability and more positive interpretations of cognitive and somatic anxiety. Mastery imagery ability was not directly associated with cognitive and somatic anxiety intensity. However, there was an indirect pathway via confidence (cognitive: *β* = −0.065, *p* = 0.006, 95% CI = −0.148 to −0.018; somatic: *β* = −0.036, *p* = 0.042, 95% CI = −0.024 to −0.063).

## Discussion

The aim of the present study was to examine the extent to which individual differences in mastery imagery ability mediated the relationship between confidence and anxiety responses immediately prior to a standardized acute psychological stress task, and the associations these variables had with subsequent performance on the task. Self-report task ratings indicated that the PASAT was an effective psychological stressor; participants reported the task as being considerably stressful and difficult. Participants also reported that they were very engaged in the task. Consequently, the laboratory paradigm was successful in creating a stressful environment to measure anxiety and performance. The hypothesized model tested is displayed in Figure 1. As displayed in Figure 2, results generally supported the hypothesized model and the important role of mastery imagery ability in both anxiety and performance during psychological stress. In support of our hypotheses, mastery imagery ability mediated the relationship between confidence and anxiety interpretation. More confident individuals were typically able to image mastery type content more easily which in turn was associated with more positive perceptions of their anxiety experienced in relation to the PASAT. As well as supporting our hypotheses, this finding supports the notion of the revised applied model of deliberate imagery use that imagery ability is a likely mediator (Cumming and Williams, 2013). Alternate model 2 further emphasized the important mediating role mastery imagery ability plays in the confidence-anxiety direction relationship, as the paths from confidence to anxiety direction in alternate model 2 were non-significant, suggesting greater confidence is only associated with more positive anxiety through greater mastery imagery ability. Although mastery imagery ability mediated the confidence-anxiety relationship for anxiety interpretation, there was no association between participants' mastery imagery ability and the intensity of their anxiety experienced. Instead, confidence directly predicted anxiety intensity.

The lack of a direct association between mastery imagery ability and anxiety intensity is somewhat surprising given that previous work has found an association between mastery imagery ability and anxiety intensity, but not between mastery imagery ability and anxiety interpretation (Quinton et al., 2018). However, an important difference between the current study and the previous work is that Quinton et al. (2018) examined trait levels of anxiety intensity and interpretation. Research examining the explicit use of mastery imagery content during stressful situations (rather than the general ability to image mastery-based content), demonstrates that mastery imagery use tends to have a greater impact on anxiety interpretation than the intensity (Williams et al., 2010; Williams and Cumming, 2012a, Williams et al., 2017). Furthermore, preliminary results have suggested that increasing mastery imagery ability through an imagery training intervention may be effective in altering anxiety interpretation rather than intensity in relation to psychological stress (Möller, 2018). Collectively, this body of work suggests that mastery imagery ability may be important for anxiety intensity at a general level, but more important for anxiety interpretation when the anxiety is in response to a specific stressful situation, event, or task.

The present findings demonstrated that the mediating role of imagery ability was similar for both cognitive and somatic anxiety. This finding is in contrast to the findings by Quinton et al. (2018) in which mastery imagery ability was associated with cognitive but not somatic anxiety. The similar results for cognitive and somatic anxiety in the present study support an argument proposed by Quinton et al. (2018) that mastery imagery ability may be more closely associated with somatic anxiety that is assessed in response to a specific situation rather than at a more general level. Irrespective of this, the results of the present study add to the growing body of work that highlights the importance of mastery imagery ability as a correlate of anxiety. Unlike the majority of work which has identified mastery imagery ability's associations with traits and dispositions, the present study demonstrates that mastery imagery ability is also associated with cognitions and emotions elicited in response to an acute psychological stress (e.g., state responses). This is important as it suggests mastery imagery ability can contribute to more facilitative responses to acute psychological stress.

It was hypothesized that mastery imagery ability would be indirectly related to performance through the proposed associations between the anxiety constructs and performance. None of the anxiety constructs were associated with performance. This may appear surprising given that theories and previous findings suggest a strong association between anxiety and performance (Weinberg and Genuchi, 1980; Hopko et al., 2003; Vitasari et al., 2010; Neil et al., 2012; Moore et al., 2013). However, anxiety is a multifaceted and complex emotion which does not always influence performance the same way. Indeed, some individuals can improve their performance when they feel more anxious, others can experience a reduction in performance, and some will experience no change in performance (Gray et al., 2013). Such variations are likely to explain the lack of association in the present study between performance and any of the anxiety constructs.

In the present study there was no relationship between anxiety and performance. However, mastery imagery ability, similar to confidence, was directly associated with performance. The direct association between mastery imagery ability and performance was also evident in the alternate models examined. Specifically, individuals who were able to more easily image themselves coping and doing well in difficult situations performed better on the acute psychological stress task. Although previous studies have demonstrated that better imagery ability can lead to greater performance benefits through imagery use (Robin et al., 2007), the present study is the first to demonstrate that higher imagery ability is also directly associated with better performance in the absence of an imagery intervention (and thus explicit imagery use). It could be that individuals better able to image are using some form of imagery as a strategy to help them perform the task (e.g., imaging the number previously heard to help remember it to add to the next number heard). Alternatively, being able to more easily see oneself in control and performing well in difficult or stressful situations may translate to actually coping and performing better in such situations. Research demonstrates that increasing movement imagery ability can improve performance through imagery use compared to those with lower imagery ability (Williams et al., 2013). Some work suggests that during imagery, compared with poor imagers, individuals with higher imagery ability experience an altered pattern of neural activation (Guillot et al., 2008) that may possibly improve performance. However, how this neural activity differs between good and poor imagers and how it influences performance has not been extensively examined. Future research is needed to demonstrate whether increasing mastery imagery ability leads to improved performance during exposure to psychological stress, and whether or not this is mediated by alternations in stressor-evoked neural activation.

Irrespective of the mechanism through which mastery imagery ability operates, the present study suggests that individuals displaying greater mastery imagery ability are better equipped with the necessary coping resources to meet the demands of stressful and/or pressurized situations, which results in better performance. From an applied point of view, the findings of the present study suggest that screening for mastery imagery ability may be a technique to help identify those individuals who may be better able to cope in stressful situations, and also those who may be more inclined to struggle and therefore need additional support to succeed. Furthermore, should mastery imagery ability be low, one way to increase it could be through enhancing self-confidence.

The present study utilized a validated laboratory acute psychological stressor. While research has demonstrated that laboratory tasks do generalize to real life stressors (e.g., Zanstra and Johnston, 2011), they are not a perfect indicator of all “real life” stress. Therefore, it is important for future research to examine the extent to which mastery imagery ability is associated with performance in more ecologically valid stress settings (e.g., athletes in pressurized sporting competitions, students during examinations). From a research point of view, it is important that future work gain a greater understanding of the mastery imagery ability and performance relationship, by continuing to investigate the psychological, biological, and neural correlates of mastery imagery ability and the effects these correlates have during stress.

Strengths of the present study included the use of path analyses rather than a series of separate regressions to more accurately examine both the hypothesized direct and indirect associations between variables, as well as the comparison with alternate models. Other strengths included the task employed and the temporal nature of the assessments. The use of assessments of confidence and anxiety that were taken immediately prior to (rather than during or after) the stress task prevented their relationship with performance being influenced by participants' evaluation of their own performance. The use of a validated psychological stress task which provided an objective measure of performance enabled us to more accurately examine the associations that confidence, anxiety, and mastery imagery ability had with performance during stressful situations. However, it is important to note that people respond differently to stress depending on the nature of the task (Trotman et al., 2018). Research should therefore re-examine the proposed model using other stressful situations in more naturalistic settings such as athletes during a pressurized sporting competition.

A limitation of the present study could be that confidence and anxiety constructs were assessed with single items and mastery imagery ability with three items. However, the IAMS has been validated against valid multi-item subscales of anxiety and confidence demonstrating high correlations between the two measures (Thomas et al., 2002). Furthermore, The SIAQ has repeatedly demonstrated good psychometric properties and internal reliability with Cronbach alpha coefficients being 0.70 or above. Despite the rigorous approach employed, the cross-sectional nature of the work prevents any conclusions of causal relationships existing between variables (Christenfeld et al., 2004). Although there is some preliminary data suggesting that improving mastery imagery ability may lead to more positive perceptions of anxiety (Möller, 2018), it is important that future research comprehensively examines the effects of increasing mastery imagery ability on anxiety responses and performance during stress. Due to the healthy population in the present study, future research should also examine the hypothesized model in clinical populations (e.g., those with a clinical diagnosis of anxiety) to determine whether the relationship between confidence, mastery imagery ability, anxiety intensity, anxiety interpretation, and performance is similar to that of a non-clinical population. In clinical populations experiencing greater anxiety levels, it could be argued that unlike the present study, the relationship between mastery imagery ability and anxiety may be more salient with intensity rather than interpretation.

In conclusion, the present study examined the extent to which mastery imagery ability mediated the relationship between confidence and anxiety responses to stress. Results demonstrated that although confidence was negatively associated with cognitive and somatic anxiety intensity, mastery imagery ability mediated the relationship between confidence and cognitive and somatic anxiety interpretation. There was no association between anxiety constructs and performance, but greater mastery imagery ability and confidence were both directly associated with better PASAT performance. Findings highlight the importance of confidence and mastery imagery ability in eliciting more facilitative interpretations of anxiety and greater performance during stress evoking situations. Future work should examine the effectiveness of increasing mastery imagery ability, and the subsequent effects this has on anxiety and performance during stress.

## Data Availability Statement

The raw data supporting the conclusions of this article will be made available by the authors, without undue reservation.

## Ethics Statement

The studies involving human participants were reviewed and approved by University of Birmingham Science, Technology, Engineering and Mathematics (STEM) Ethical Review Committee. The patients or participants provided their written informed consent to participate in this study.

## Author Contributions

SW made substantial contributions to the study design, developed the model to be tested, conducted the data analysis, and drafted the manuscript. MQ assisted in the model to be tested, and provided substantial critical analysis of, and feedback on the manuscript. JVvZ made substantial contributions to the study design, and provided critical analysis of, and feedback on the manuscript. JD and CM made substantial contributions to data collection and entry, and provided critical analysis of, and feedback on the manuscript. GT made substantial contributions to the study design, data collection and entry, and provided feedback on the manuscript. AG contributed to the study design, provided critical analysis of, and feedback on the model to be tested, and provided substantial critical analysis of, and feedback on the manuscript. All authors contributed to the article and approved the submitted version.

## Conflict of Interest

The authors declare that the research was conducted in the absence of any commercial or financial relationships that could be construed as a potential conflict of interest.

## Footnotes

^1^For a detailed description of these tasks, please see Trotman et al. (2019).

## References

[B1] Bandura A. (1997). Self-Efficacy: The Exercise of Control. New York, NY: W.H. Freeman.

[B2] Bentler P. M. (1995). EQS Structural Equations Program Manual. Encino, CA: Multivariate Software Inc.

[B3] Blascovich J. Mendes W. B. (2000). Challenge and threat appraisals. The role of affective cues, in Feeling and Thinking: The Role of Affect in Social Cognition, 1st Edn., ed Forgas J. P. (New York: Cambridge University Press), 59–82.

[B4] Carrier C. Higson V. Klimoski V. Peterson E. (2014). The effects of facilitative and debilitative achievement anxiety on notetaking. J. Educ. Res. 77, 133–138. 10.1080/00220671.1984.10885512

[B5] Chamberlain S. T. Hale B. D. (2007). Competitive state anxiety and self-confidence: intensity and direction as relative predictors of performance on a golf putting task. Anxiety Stress Coping 20, 197–207. 10.1080/10615800701288572 17999224

[B6] Christenfeld N. J. Sloan R. P. Carroll D. Greenland S. (2004). Risk factors, confounding, and the illusion of statistical control. Psychosom. Med. 66, 868–875. 10.1097/01.psy.0000140008.70959.41 15564351

[B7] Cohen S. (1980). Aftereffects of stress on human performance and social behaviour: a review of research and theory. Psychol. Bull. 88, 82–108. 10.1037/0033-2909.88.1.82 7403392

[B8] Crum A. J. Akinola M. Martin A. Fath S. (2017). The role of stress mindset in shaping cognitive, emotional, and physiological responses to challenging and threatening stress. Anxiety Stress Coping 30, 379–395. 10.1080/10615806.2016.1275585 28120622

[B9] Cumming J. Olphin T. Law M. (2007). Self-reported psychological states and physiological responses to different types of motivational general imagery. J. Sport Exerc. Psychol. 29, 629–644. 10.1123/jsep.29.5.629 18089896

[B10] Cumming J. Williams S. E. (2013). Introducing the revised applied model of deliberate imagery use for sport, dance, exercise, and rehabilitation. Mov. Sport Sci. 82, 69–81. 10.3917/sm.082.0069

[B11] Ginty A. T. Gianaros P. J. Derbyshire S. W. G. Phillips A. C. Carroll D. (2013). Blunted cardiac stress reactivity relates to neural hypoactivation. Psychophysiology 50, 219–229. 10.1111/psyp.12017 23351027

[B12] Ginty A. T. Phillips A. C. Higgs S. Heaney J. L. J. Carroll D. (2012). Disordered eating is associated with blunted cortisol and cardiovascular reactions to acute psychological stress. Psychoneuroendocrinology 37, 715–724. 10.1016/j.psyneuen.2011.09.004 21962379

[B13] Gray R. Allsop J. Williams S. E. (2013). Changes in putting kinematics associated with choking and excelling under pressure. Int. J. Sport Psychol. 44, 387–407. 10.7352/IJSP2013.44.387

[B14] Gronwall D. (1977). Paced auditory serial-addition task: a measure of recovery from concussion. Percept. Mot. Skills 44, 367–373. 10.2466/pms.1977.44.2.367 866038

[B15] Guillot A. Collet C. Nguyen V. A. Malouin F. Richards C. Doyon J. (2008). Functional neuroanatomical networks associated with expertise in motor imagery. NeuroImage 41, 1471–1483. 10.1016/j.neuroimage.2008.03.042 18479943

[B16] Hanton S. Jones G. (1999). The acquisition and development of cognitive skills and strategies: I. Making the butterflies fly in formation. Sport Psychol. 13, 1–21. 10.1123/tsp.13.1.1

[B17] Hanton S. Mellalieu S. D. Hall R. (2004). Self-confidence and anxiety interpretation: a qualitative investigation. Psychol. Sport Exerc. 5, 477–495. 10.1016/S1469-0292(03)00040-212430992

[B18] Hayes A. F. (2013). Introduction to Mediation, Moderation, and Conditional Process Analysis: A Regression-Based Approach. New York, NY: The Guilford Press.

[B19] Hopko D. R. NcNeil D. W. Lejuez C. W. Ashcraft M. H. Eifert G. H. Riel J. (2003). The effects of anxious responding on mental arithmetic and lexical decision task performance. J. Anxiety Disord. 17, 647–665. 10.1016/S0887-6185(02)00240-2 14624816

[B20] Hu L. T. Bentler P. M. (1999). Cutoff criteria for fit indexes in covariance structure analysis: conventional criteria versus new alternatives. Struct. Equat. Model. 6, 1–55. 10.1080/10705519909540118

[B21] Jamieson J. P. Peters B. J. Greenwood E. J. Altose A. J. (2016). Reappraising stress arousal improves performance and reduces evaluation anxiety in classroom exam situations. Soc. Psychol. Person. Sci. 7, 579–587. 10.1177/1948550616644656

[B22] Johnston D. W. Tuomisto M. T. Patching G. R. (2008). The relationship between cardiac reactivity in the laboratory and in real life. Health Psychol. 27, 34–42. 10.1037/0278-6133.27.1.34 18230011

[B23] Jones G. Hanton S. (2001). Cognitive labeling of precompetitive affective states as a function of directional anxiety interpretations. J. Sports Sci. 19, 385–395. 10.1080/026404101300149348 11411775

[B24] Jones G. Swain A. (1995). Predispositions to experience debilitative and facilitative anxiety in elite and non-elite performance. Sport Psychol. 9, 201–211. 10.1123/tsp.9.2.201

[B25] Jones M. V. Meijen C. McCarthy P. J. Sheffield D. (2009). A theory of challenge and threat states in athletes. Int. Rev. Sport Exerc. Psychol. 2, 161–180. 10.1080/17509840902829331 32116930 PMC7016194

[B26] Jöreskog K. G. Sörbom D. (1993). LISREL 8 User's Reference Guide. Chicago, IL: Scientific Software.

[B27] Lang P. J. (1979). A bio-informational theory of emotional imagery. Psychophysiology 16, 495–512. 10.1111/j.1469-8986.1979.tb01511.x 515293

[B28] Lazarus R. S. Deese J. Osler S. F. (1952). The effects of psychological stress upon performance. Psychol. Bull. 49, 293–317. 10.1037/h0061145 12983450

[B29] Martens R. Vealy R. S. Burton D. (1990). Competitive Anxiety in Sport. Champaign, IL: Human Kinetics.

[B30] Mellalieu S. D. Hanton S. Thomas O. (2009). The effects of a motivational general-arousal imagery intervention upon preperformance symptoms in male rugby union players. Psychol. Sport Exerc. 10, 175–185. 10.1016/j.psychsport.2008.07.003

[B31] Möller C. M. (2018). The Effect of Mastery Imagery Ability on Appraisals and Responses to Stress. Unpublished master's thesis, University of Birmingham, Birmingham, UK. 31396128

[B32] Moore L. J. Wilson M. R. Vine S. J. Coussens A. H. Freeman P. (2013). Champ or chump?: Challenge and threat states during pressurized competition. J. Sport Exerc. Psychol. 35, 551–562. 10.1123/jsep.35.6.551 24334317

[B33] Morris L. Davis D. Hutchings C. (1981). Cognitive and emotional components of anxiety: literature review and revised worry-emotionality scale. J. Educ. Psychol. 75, 541–555. 10.1037/0022-0663.73.4.541 7024371

[B34] Neil R. Wilson K. Mellalieu S. D. Hanton S. Taylor J. (2012). Competitive anxiety intensity and interpretation: a two-study investigation into their relationship with performance. Int. J. Sport Exerc. Psychol. 10, 96–111. 10.1080/1612197X.2012.645134

[B35] Quinton M. L. Cumming J. Williams S. E. (2018). Investigating the mediating role of positive and negative mastery imagery ability. Psychol. Sport Exerc. 35, 1–9. 10.1016/j.psychsport.2017.10.011

[B36] Quinton M. L. Veldhuijzen van Zanten J. J. C. S. Trotman G. P. Cumming J. Williams S. E. (2019). Investigating the protective role of mastery imagery ability in buffering debilitative stress responses. Front. Psychol. 10:1657. 10.3389/fpsyg.2019.01657 31396128 PMC6668598

[B37] Ring C. Burns V. E. Carroll D. (2002). Shifting hemodynamics of blood pressure control during prolonged mental stress. Psychophysiology 39, 585–590. 10.1111/1469-8986.3950585 12236324

[B38] Robin N. Dominique L. Toussaint L. Blandin Y. Guillot A. Le Her M. (2007). Effect of motor imagery training on service return accuracy in tennis: the role of imagery ability. Int. J. Sport Exerc. Psychol. 2, 175–186. 10.1080/1612197X.2007.9671818

[B39] Robinson S. J. Leach J. Owen-Lynch P. J. Sünram-Lea S. I. (2013). Stress reactivity and cognitive performance in a simulated firefighting emergency. Aviat. Space Environ. Med. 84, 592–599. 10.3357/ASEM.3391.2013 23745287

[B40] Seipp B. (1991). Anxiety and academic performance: a meta-analysis of findings. Anxiety Res. 4, 27–41. 10.1080/08917779108248762

[B41] Skinner N. Brewer N. (2004). Adaptive approaches to competition: challenge appraisals and positive emotion. J. Sport Exerc. Psychol. 26, 283–305. 10.1123/jsep.26.2.283

[B42] Swain A. Jones G. (1996). Explaining performance variance: the relative contribution of intensity and direction dimensions of competitive state anxiety. Anxiety Stress Coping 9, 1–18. 10.1080/10615809608249389

[B43] Tabachnick B. G. Fidell L. S. (2013). Using Multivariate Statistics. Essex: Pearson Education Limited.

[B44] Thomas O. Hanton S. Jones G. (2002). An alternative approach to short-form self report assessment of competitive anxiety. Int. J. Sport Psychol. 33, 325–336.

[B45] Thomas O. Maynard A. Hanton S. (2004). Temporal aspects of competitive anxiety and self-confidence as a function of anxiety perceptions. Sport Psychol. 18, 172–187. 10.1123/tsp.18.2.172

[B46] Trotman G. P. Veldhuijzen van Zanten J. J. C. S. Davies J. Möller C. Ginty A. T. Williams S. E. (2019). Associations between heart rate, perceived heart rate, and anxiety during psychological stress. Anxiety Stress Coping 5, 1–17. 10.1080/10615806.2019.1648794 31382769

[B47] Trotman G. P. Williams S. E. Quinton M. L. Veldhuijzen Van Zanten J. J. C. S. (2018). Challenge and threat states: examining cardiovascular, cognitive and affective responses to two distinct laboratory stress tasks. Int. J. Psychophysiol. 126, 42–51. 10.1016/j.ijpsycho.2018.02.004 29477547

[B48] Veldhuijzen van Zanten J. J. Ring C. Burns V. E. Edwards K. M. Drayson M. Carroll D. (2004). Mental stress-induced hemoconcentration: sex differences and mechanisms. Psychophysiology 41, 541–551. 10.1111/j.1469-8986.2004.00190.x 15189477

[B49] Vitasari P. Wahab M. N. A. Othman A. Herawan T. Sinnadurai S. K. (2010). The relationship between study anxiety and academic performance among engineering students. Proc. Soc. Behav. Sci. 8, 490–497. 10.1016/j.sbspro.2010.12.06731879453

[B50] Weinberg R. S. Genuchi M. (1980). Relationship between competitive trait anxiety, state anxiety, and golf performance: a field study. J. Sport Psychol. 2, 148–154. 10.1123/jsp.2.2.148

[B51] Willemsen G. Ring C. Carroll D. Evans P. Clow A. Hucklebridge F. (1997). Secretory immunoglobulin A and cardiovascular reactions to mental arithmetic and cold pressor. Psychophysiology 35, 252–259. 10.1111/1469-8986.3530252 9564745

[B52] Williams S. Quinton M. Veldhuijzen van Zanten J. Davies J. Moller C. Trotman G.. (2019). The benefits of mastery imagery ability during acute psychological stress, Paper presented at the 40th International Conference of the Stress and Anxiety Research Society (Palma).

[B53] Williams S. E. Carroll D. Veldhuijzen van Zanten J. J. C. S. Ginty A. T. (2016). Anxiety symptom interpretation: a potential mechanism explaining the cardiorespiratory fitness-anxiety relationship. J. Affect. Disord. 193, 151–156. 10.1016/j.jad.2015.12.051 26773908

[B54] Williams S. E. Cooley S. J. Cumming J. (2013). Layered stimulus response training improves motor imagery ability and movement execution. J. Sport Exerc. Psychol. 35, 60–71. 10.1123/jsep.35.1.60 23404880

[B55] Williams S. E. Cumming J. (2011). Measuring athlete imagery ability: the Sport Imagery Ability Questionnaire. J. Sport Exerc. Psychol. 33, 416–440. 10.1123/jsep.33.3.416 21659671

[B56] Williams S. E. Cumming J. (2012a). Challenge vs. threat: investigating the effect of using imagery to manipulate stress appraisal of a dart throwing task. Sport Exerc. Psychol. Rev. 8, 4–21.

[B57] Williams S. E. Cumming J. (2012b). Sport imagery ability predicts trait confidence, and challenge and threat appraisal tendencies. Eur. J. Sport Sci. 12, 499–508. 10.1080/17461391.2011.630102

[B58] Williams S. E. Cumming J. (2015). Athlete imagery ability: a predictor of confidence and anxiety intensity and direction. Int. J. Sport Exerc. Psychol. 14, 268–280. 10.1080/1612197X.2015.1025809

[B59] Williams S. E. Cumming J. Balanos G. M. (2010). The use of imagery to manipulate challenge and threat appraisal states in athletes. J. Sport Exerc. Psychol. 32, 339–358. 10.1123/jsep.32.3.339 20587822

[B60] Williams S. E. Veldhuijzen van Zanten J. J. C. S. Trotman G. P. Quinton M. L. Ginty A. T. (2017). Challenge and threat imagery manipulates heart rate and anxiety responses to stress. Int. J. Psychophysiol. 117, 111–118. 10.1016/j.ijpsycho.2017.04.011 28461204

[B61] Zanstra Y. J. Johnston D. W. (2011). Cardiovascular reactivity in real life settings: measurement, mechanisms and meaning. Biol. Psychol. 86, 98–105. 10.1016/j.biopsycho.2010.05.002 20561941 PMC3131085

